# Infectious disease outbreak preparedness and response in Nigeria: history, limitations and recommendations for global health policy and practice

**DOI:** 10.3934/publichealth.2020057

**Published:** 2020-09-22

**Authors:** Testimony J Olumade, Oluwafolajimi A Adesanya, Iyanuoluwa J Fred-Akintunwa, David O Babalola, Judith U Oguzie, Olusola A Ogunsanya, Uwem E George, Oluwawapelumi D Akin-Ajani, Damilola G Osasona

**Affiliations:** 1 African Centre of Excellence for Genomics of Infectious Diseases, Redeemer's University, Ede, Osun, Nigeria; 2 Department of Biological Sciences, Redeemer's University, Ede, Osun, Nigeria; 3 Department of Medicine, College of Medicine, University of Ibadan, Ibadan, Nigeria; 4 Department of Veterinary Pathology, Faculty of Veterinary Medicine, University of Ibadan, Ibadan, Nigeria

**Keywords:** infectious diseases, outbreak preparedness, outbreak response, global health policy, Nigeria

## Abstract

Effective disease outbreak response has historically been a challenging accomplishment for the Nigerian health system due to an array of hurdles not unique to Nigeria but also found in other African nations which share its large size and complexity. However, the efficiency of the response mounted against the Ebola Virus Disease (EVD) outbreak of 2014 proved that indeed, though challenging, proactive and effective outbreak response is not impossible. With over 20 public health emergencies and infectious disease outbreaks between 2016 and 2018 alone, Nigeria is one of only five members of the World Health Organization (WHO) African Region to report five or more public health events per annum. There are many lessons that can be drawn from Nigeria's experience in handling outbreaks of infectious diseases. In this review, we discuss the history of emerging and re-emerging infectious disease outbreaks in Nigeria and explore the response strategies mounted towards each. We also highlight the significant successes and note-worthy limitations, which we have then utilized to proffer policy recommendations to strengthen the Nigerian public health emergency response systems.

## 1. Introduction

Often referred to as the most populous African country, with a population of over 200 million [1], Nigeria has been faced with numerous challenges in its health sector for several years. With a dearth of effective policy implementation coupled with the burden of infrastructural decay and lack of political will, the health sector has seen little development reminiscent of the standards required of national health systems in the 21st century. This has translated into a handicapping situation, towards the preparedness and response of the country to infectious disease outbreaks in recent times. The Federal Ministry of Health (FMoH) had earlier constituted the National Emergency Response and Preparedness Team in a move that acknowledged the importance of effective infectious disease outbreak prevention, response, and control [2]. This has, however, done little to stem the recurrent tides of such outbreaks, and the devastating effects they have on both human lives and economic capital of the nation. In order to develop a holistic national outbreak preparedness and response plan, there is a need to review the response to previous outbreaks in recent time, highlighting the positives and underscoring the challenges, for the purpose of making timely policy recommendations that can be translated into practice for the advancement of global health and to improve responses to future outbreaks of infectious diseases in the country; hence the aim of this review. In summary, following a review of the available evidence, we found Lassa fever, Monkey Pox, Ebola Virus Disease, Yellow Fever, and Poliomyelitis to be the top five emerging and re-emerging infectious diseases outbreaks Nigeria has battled since the turn of the century. While the establishment of the Nigeria Center for Disease Control (NCDC) was a positive important turning point in Nigeria's fight against infectious diseases outbreaks, factors such as poor healthcare funding, inadequate diagnostic capacity, political instability, insecurity and personnel shortage, continue to limit the ability of the Nigerian public healthcare system to effectively prepare and respond to infectious disease outbreaks.

This review was conducted by searching for literature on the topic across several databases—PubMed, Google Scholar, EMBASE, and grey literature (official documents). Keywords were “Preparedness”, “Infectious Disease”, “Outbreak”, “Response”, and “Nigeria”. Full texts of the articles retrieved from all databases were imported into Mendeley Reference Management Software and organized to address the different sub-headings of the review. They were screened for access to the full text and articles with full texts available were used to inform the narrative review of the literature.

## 2. History of emerging and re-emerging infectious diseases outbreaks in Nigeria

Emerging infectious diseases are diseases that either have never occurred in humans before, previously occurred but only affecting a limited number of people in affected places, or have occurred throughout history but only recently identified as distinctly due to an infectious agent while re-emerging infectious diseases are those that were once major public health problems globally for a significant portion of the population.

### 2.1. Lassa fever (LF)

LF is an acute viral hemorrhagic fever caused by the single-stranded RNA virus from the family *Arenaviridae* [3]. It has been said that 80% of infected individuals remain asymptomatic with others displaying acute symptoms of fever, weakness, chest pain, and gastrointestinal (GI) side effects such as nausea, vomiting and diarrhea [4]. About 1–15% of symptomatic cases present with severe symptomatology ranging from: abnormal bleeding from the facial orifices, hearing loss, tremors, encephalitis, coma and death [5]. With an incubation period of 6 to 21 days [6], LF has been known to be associated closely with seasonal variations, peaking in the dry season usually between the months of December and April [7], a situation attributed to the migration of the rodent reservoirs into human settlements in search for food. LF is one infectious disease that has caused emerging and re-emerging outbreaks in Nigeria. Since its discovery in Lassa, Borno State, in 1969 [8], there have been outbreaks in Jos, Plateau (1969–1970); Zonkwa, Kaduna (1974); Pankshin, Plateau and Onitsha, Anambra (1976); Ekpoma (1989); Lafia (1993–1994); Ebonyi and Ogun (2005) after which subsequent outbreaks involved multiple states across several geopolitical zones [7],[9]. The epidemic of 2011–2012 is regarded as the worst outbreak so far, affecting 41 local government areas (LGAs) in 23 states, resulting in 937 cases and 95 deaths—a case: fatality rate (CFR) of 10.14% [7]. This statistic worsened in 2018 [10] and now seems to have further worsened in 2020, with 3,735 suspected cases, of which 906 are laboratory-confirmed as of March 15, 2020 [11]. Thus, LF remains one infectious disease that has re-emerged several times over and continues to pose a significant challenge to the Nigerian health system.

### 2.2. Ebola virus disease (EVD)

EVD is an acute viral hemorrhagic fever caused by the Ebola virus, which belongs to the family *Filoviridae* [12]. The first case of EVD in Nigeria was confirmed on July 25, 2014 [13], triggering an outbreak infecting 20 people and killing 8 (CFR = 40%) [13]. The Nigerian outbreak was brought under control in record time, with the World Health Organization (WHO) declaring the nation free from Ebola transmission on October 20, 2014 [14],[15].

### 2.3. Yellow fever disease (YFD)

YFD is an acute viral hemorrhagic fever caused by the yellow fever virus, a member of the family *Flaviviridae*, and transmitted by an infected female Aedes mosquito The first recorded yellow fever outbreak in Nigeria occurred in Lagos in 1864. This was followed by other outbreaks in Lagos in 1894, 1905, 1906, 1925 and 1926 [16]. The next outbreak happened in Jos in 1969, infecting over 100,000 people, and then in 1987 and 1996, infecting over 120,000 people in different parts of the country including: Jos, Azare in Bauchi state, Ogoja in Cross River state, Oju in Benue state and Ogbomosho in Oyo state [17]. There were only sporadic cases after this period and for 21 years, till September 2017, when a 7-year old presented with the classic symptoms of yellow fever in the Ifelodun LGA of Kwara state [18]. This eventually resulted in an outbreak of 4,189 suspected cases and 604 confirmed nationwide. Following the 2017 outbreak, the nation has fought yearly outbreaks since then, in 2018 [19], 2019 [20] and 2020 [21].

### 2.4. Poliomyelitis

This is a highly infectious viral disease caused by the poliovirus, which belongs to the family of Enteroviruses and results in muscle weakness or paralysis of the limbs, with very few other symptoms except minor headaches, neck stiffness, and stiffness of the arms and legs [22]. The last major outbreak to happen in Nigeria was in 2007, involving 69 children in Northern Nigeria, and was a direct consequence of the refusal of locals to vaccinate their children due to anti-vaccination religious propaganda [23]. In 2015, the WHO removed Nigeria from the list of polio-endemic nations due to the high likelihood that wild poliovirus (WPV) circulation had been interrupted in Nigeria. However, four new cases were discovered in August and September 2016 in Borno, and were attributed to the destabilization of healthcare infrastructure as a consequence of the protracted Boko Haram insurgency in North-East Nigeria. Following this isolated occurrence, no new case has been reported to date [24].

### 2.5. Monkeypox disease

The monkeypox virus was first identified in 1958 in captive monkeys imported into Copenhagen, Denmark from Africa [25]. However, the first human case of monkeypox was identified in a 9-year old in the village of Bukenda, Zaire (now the Democratic Republic of Congo (DRC)) [26]. Between 1970 and 1978, Nigeria reported a total of 3 cases of human monkeypox infection, one in 1970 and two in 1978 [26], and none until 38 years later, in September 2017, when a re-emergence of what would be the largest ever recorded outbreak of the West African Clade of human monkeypox, with 228 suspected cases (and 60 confirmed) in 24 of 36 states in the country [27]. The index case was an 11-year old boy referred to the Niger Delta University Teaching Hospital (NDUTH) with symptoms suggestive of chickenpox. However, this was later ruled out due to the nature of the associated skin lesions and persistence of symptoms, and monkeypox was then considered as a possible diagnosis [28].

## 3. Some of Nigeria's outbreak preparedness, detection, and response efforts so far

In response to the elaborate history of infectious disease outbreaks in Nigeria, the idea of a national public health institute charged with the responsibility of preventing, detecting, and responding to infectious disease threats in the country was first conceived in 2007; however, it was not until 2011 that the Nigerian Centre for Disease Control (NCDC) was established by the amalgamation of certain instruments of the Federal Ministry of Health (FMoH)—Epidemiology Division, Avian Influenza Project and the Nigeria Field Epidemiology and Laboratory Training Program (NFELTP) [29]. Retrospectively speaking, this singular act represents one of the most important efforts taken towards preparedness against infectious disease outbreaks in the nation, as would be highlighted shortly. Since its establishment, the NCDC has grown rapidly into its role and the achievement of its mandate to lead the preparedness, detection and response to infectious disease outbreaks and public health emergencies in the country [30]. Through the establishment of a nationwide network of reference laboratories, which has matured into a consortium of five viral hemorrhagic fever (VHF) laboratories, four yellow fever/measles/rubella laboratories, 17 cholera/cerebrospinal meningitis laboratories and four sentinel sites each for influenza and hepatitis E/Rotavirus, the NCDC has strengthened the diagnostic capabilities of the Nigerian health system, in preparation for potential infectious diseases outbreaks [30].

Furthermore, through the NFELTP, a 2-year in-service training in applied epidemiology and laboratory techniques, implemented in partnership with the African Field Epidemiology Network, the NCDC is building a critical mass of highly-skilled indigenous public health specialists who can be deployed to curtail an infectious disease outbreak should any arise [31],[32]. The program has developed a pool of field epidemiologists who have been instrumental in coordinating the response to several infectious disease outbreaks in recent times, most notable was their role in the effective response to the 2014 outbreak of EVD, a feat that was rightly described by the WHO as “a piece of world-class epidemiological detective work” [33]. In order to further deepen its ability to coordinate preparedness and response activities, the NCDC established an Incident Coordination Centre (ICC), to review reports from previous and current outbreaks and map out holistic plans towards preparedness for future outbreaks and containment efforts for on-going ones [29].

The NCDC has also forged sustainable partnerships with foreign bilateral and multilateral agencies such as the US Centers for Disease Control and Prevention (CDC), the WHO, and the ECOWAS Regional Centre for Disease Control, which support the work of the agency via several grants and technical assistance to support infectious disease surveillance, establish a working laboratory network for diagnosis and other outbreak and disaster response activities [30]. It remains a fact that the NCDC has taken giant strides towards the achievement of its mandate, in this paper we dive into the specifics, examining the practical steps the agency has taken to prepare for, detect and respond to infectious disease outbreaks in the country in recent times.

### 3.1. Ebola virus disease (EVD): July 2014–October 2014

The spread of the EVD to Nigeria in 2014 was accompanied by a great deal of concern locally and internationally, enough to instigate the Director-General of the WHO to declare a Public Health Emergency of International Concern (PHEIC) [15]. This is because the outbreak affected two of the largest complex megacities in the country—Lagos and Port Harcourt, with a combined population of over 30 million people, of which a significant proportion live in crowded slums and shanties with limited public health-supportive infrastructure. There were concerns about the possibility of effective contact tracing under such circumstances and the situation was given numerous elaborate appellations such as “powder keg situation”, “potential apocalyptic urban outbreak” and “potentially the most explosive Ebola outbreak imaginable” [33], but indeed the reality of what ensued was a stark contrast to all projections that had been made. Barely three months from when the first case was established, Nigeria had fulfilled the 42 days benchmark needed to be declared free of Ebola virus transmission by the WHO, with 20 cases and 8 deaths [13],[14].

But how did this happen? A 2016 report by Musa et al. [34] documented the robust detection and response mechanisms deployed by all involved stakeholders, which ensured that the outbreak was effectively curtailed. The EVD outbreak response in Nigeria was led by the NCDC, in collaboration with the Ministries of Health (MoH) of Lagos and Rivers State, utilizing existing integrated disease surveillance and response (IDSR) systems to ensure effective contact tracing, rapid identification of suspected cases, laboratory diagnoses to establish confirmed cases and clinical management of all cases. The response also involved strategies for safeguarding the points of entry (PoE), managing rumors and alerts from the populace as well as creating awareness and mobilizing support and goodwill from the general public [34].

**Figure 1.**
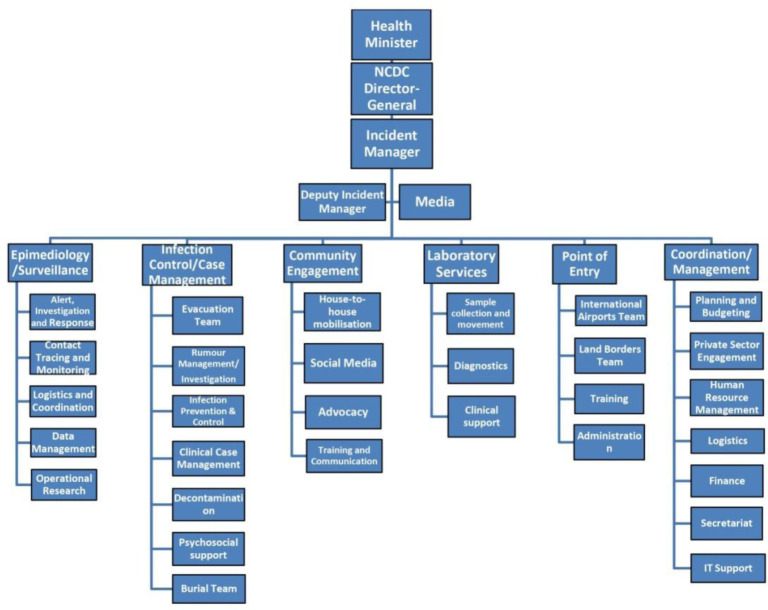
Ebola Emergency Operations Center (EOC) Organogram.

For effective response coordination, the NCDC established Ebola Emergency Operations Centers (EOC) in both affected cities, and an Incident Management System (IMS) was introduced at the forefront of outbreak response [34],[35]. The IMS was overseen by an Incident Manager (IM) (**Figure 1**), who reported directly to the NCDC, and the IMS was organized into six response teams including (i) Epidemiology/Surveillance (comprising rumor/alert management, contact tracing, data management), (ii) Case Management/Infection Control (comprising clinical management of EBV cases, infection prevention and control for health workers, psychosocial support, decontamination, and burial sub-teams), (iii) Social Mobilization, (iv) Laboratory Services, (v) Points of Entry and (vi) Management/Coordination (comprising human resources, administrations, finance, logistics and procurement [36]. Each operational team had a team leader and was semi-autonomous, being responsible for developing their own lists of staff, material and resources, and operating procedures, subject to the approval of a technical strategy group, chaired by the IM [37].

Contact tracing was conducted according to a national guideline adopted from a WHO reference document [38], by residents of the NFELTP, epidemiologists from the NCDC, Lagos and Rivers State MoH as well as WHO surveillance officers. A total of 892 contacts of interest were identified, comprising of 362 contacts in Lagos and 530 contacts in Port Harcourt [34], and contact tracers made use of android mobile phone applications with GIS capabilities to monitor daily temperature measurements of each contact while mapping their locations against their registered residential address. Contacts were reclassified as suspected cases once they met certain pre-determined criteria, and they were counseled on social distancing for a period of 21 days [34]. The temperature data from the mobile phone applications were monitored closely at the EOC, ensuring that all contacts were effectively monitored and the possibility of false data reporting by the contact tracers was eliminated. Through this innovative technique, the NCDC was able to monitor 100% of all contacts in Lagos and 99.8% of contacts in Port Harcourt, a feat that shocked the public health community, worldwide [33]. While there were a few contacts who proved challenging to follow-up and even escaped follow-up, special intervention teams were in place to track them down and bring them back into the system.

For case management, Ebola Treatment Centers (ETC) were established in both affected cities. In Lagos, a 40-bed facility (with a surge capacity of 10 beds) was set-up, while in Port Harcourt, a 26-bed facility (with a surge capacity of 8 beds) was established by the state MoH with support from the NCDC. Both facilities were managed by a combined team of Nigerian health workers, with support from the WHO and Médicins Sans Frontières (MSF) staff [34]. The ETCs were equipped with medicine, consumables, personal protective equipment (PPEs), body bags and independent ambulances. Laboratory diagnosis of confirmed cases was carried out at the Virology laboratory of the Lagos University Teaching Hospital (LUTH) and through a mobile laboratory donated by the CDC and equipped with reverse transcriptase-polymerase chain reaction (RT-PCR) capabilities [33]. An important note of preparedness to highlight at this point is the fact that the necessary primers and buffers required to make a laboratory diagnosis of EBV were available at the LUTH virology laboratory prior to the arrival of the first case. This was very instrumental to the timely diagnosis of this index case and thus the rapid deployment of detection and response efforts. For alert/rumour management, Toll-free lines were opened and widely circulated through print/electronic/social media to the public, and they were encouraged to call in to report suspected cases or to clear up any inquiries. Further, printed fliers containing important information about EVD was distributed house-to-house by trained public health personnel [34].

Community engagement/sensitization was achieved by meeting with community gatekeepers such as traditional rulers, religious leaders, youth organizations, market women associations and school administrators. Through such a systematic, holistic and well-implemented approach, EVD was curtailed in Nigeria in record time, a feat that will go down in history as an unprecedented win for an apparently struggling public health system.

Challenges encountered by the NCDC in the containment of the EVD outbreak of 2014 included a lack of infrastructure during the first few days of the outbreak. In addition, there was an on-going industrial action by medical doctors in Nigeria, making it difficult to recruit qualified personnel for clinical case management during the early days of the outbreak. [14]. Other challenges faced by the NCDC with the containment of the EVD 2014 outbreak were related to misconceptions and myths about the disease, and self-protective behavior among health workers.

### 3.2. Lassa fever: endemic

Since its discovery in 1969, Lassa fever remains endemic in Nigeria, exhibiting seasonal variation, with most cases occurring during the late rainy and early dry seasons, often due to a reduction in rain output and subsequent shortage of food for the disease vectors—multimammate rats belonging to the *Mastomys* species-complex in their natural habitat [39]. This results in their migration to human habitats, where they come into contact with humans, ultimately leading to disease transmission. One such outbreaks in Abakaliki, Ebonyi State between January to March 2012, reported by Ajayi et al. [40] highlighted significant shortcomings in outbreak preparedness, detection, and response, especially in resource-limited settings. Such shortcomings included: the lack of an infectious disease isolation ward in the tertiary health facility where the index patient was diagnosed; logistical limitations which prevented effective and timely testing of suspected cases as the reference laboratory where tests were carried out was located 300 km away from the hospital, a factor that contributed to the delayed onset of treatment for the index patient, contributing to her eventual death and forcing healthcare personnel to resort to the empirical treatment of suspected cases with ribavirin; and a shortage of necessary PPE, which was partly responsible for the nosocomial spread of the disease among healthcare workers [40].

The outbreak response was coordinated by the Lassa fever technical committee of the Federal Teaching Hospital, Abakaliki, with support from the State and Federal MoH [40]. Active disease surveillance was carried out in partnership with community elements, by several public advocacy and community mobilization efforts using print and electronic media and community gatekeepers. Contact tracing eventually identified 20 cases (10 suspected and 10 confirmed). The suspected cases could not be confirmed due to logistical challenges associated with laboratory testing unique to resource-limited settings [40]. Ultimately, the outbreak was curtailed due to the early detection of the index case, a factor attributed to the high index of suspicion of the managing team; as well as the strong collaboration between different sectors and stakeholders involved in the response.

A recent communication by Dan-Nwafor et al. (2019), described the measures taken by the NCDC to control the 2019 outbreak that occurred in 21 states between January and April 2019, a period during with 554 laboratory-confirmed cases and 124 deaths were reported. In preparation for the outbreak, national guidelines on the treatment of Lassa fever patients had been reviewed and updated 2 months earlier, to include vital infection prevention and control (IPC) measures, as well as care of complications such as septic shock and kidney injury, after Lassa fever infection [42]. This was in response to an observational cohort study [43], which showed that such complications are an important risk factor for fatality in Lassa fever patients. In addition, vital PPEs needed by frontline healthcare workers had been distributed to the Lassa fever treatment centers in anticipation of the outbreak [41].

Following the onset of the outbreak, the response involved the organization of national and zonal workshops on the clinical management of Lassa fever patients, for healthcare workers as well as community sensitization efforts for the public through print, social and electronic media channels on the mode of presentation of the disease, available treatment sites and toll-free lines for alerts and inquiries. The establishment of the Lassa fever reference laboratory network also greatly enhanced diagnostic capacity during the outbreak [41].

### 3.3. Monkey pox: September 2017–December 2017

Following this presumptive diagnosis two days after admission, hospital, state and national authorities were notified for confirmation and further investigation [25]. The diagnosis was confirmed and a multiagency interdisciplinary EOC was activated at the NCDC [26]. A week later, a Rapid Response Team (RRT) from the NCDC was deployed to Bayelsa state, to partner with the state government to respond to the outbreak and the NDUTH was subsequently designated as the treatment center for all monkeypox cases during the outbreak [25]. The NCDC prepared the Interim National Guidelines to guide the outbreak response and ensure it is carried out in a coordinated fashion [44]. At the onset of the outbreak, the NDUTH did not have a dedicated isolation ward for the monkeypox patients, compelling the hospital administration to convert the 12-bed medical ward into an isolation ward for adult cases [25].

An NDUTH monkeypox response committee was also constituted to include case management teams, waste management, and laboratory technicians, to be responsible for coordinating the hospital's response to the outbreak, with support from the NCDC and the State Ministry of Health [25]. The response committee organized hospital-wide sensitization training for all staff on IPC strategies, monkeypox case management and use of PPEs. Laboratory confirmation of suspected cases was done through real-time PCR, IgM serology and genome sequencing, and was carried out at the Institut Pasteur, Dakar, Senegal; African Centre of Excellence for Genomics of Infectious Diseases (ACEGID), Ede, Nigeria and the CDC, Atlanta, GA, USA. Further tests were carried out at the NCDC Reference Laboratory, Abuja [27]. The lack of laboratory testing facilities at NDUTH posed significant constraints on the rapid testing of suspected cases as they presented to the facility [25].

### 3.4. Yellow fever: endemic since September 2017

In September 2017, a 7-year old presented with the classic symptoms of yellow fever in the Ifelodun LGA of Kwara state. Following the confirmation of this first case, within a week, the NCDC deployed a multiagency RRT to Kwara state, to respond to the outbreak [18]. The functions of this RRT were to support the state surveillance team in conducting yellow fever surveillance activities such as verbal autopsy (getting enough information from a deceased individual to determine the cause of death), entomological surveys, reactive vaccination campaigns, and an assessment of immunization profiles of children aged 1–10 years in the affected communities [18]. The RRT was also responsible for developing the request for vaccines to be sent to the International Coordinating Group (ICG), and also to support the risk assessment and social mobilization efforts of the state government [18]. The onset of the outbreak in Nigeria followed news of similar outbreaks in other African countries such as Angola and the DRC [28]. The response of the NCDC to the outbreak may be conceptualized to be divided into the following stages: active case search, rapid yellow fever vaccination coverage assessment, verbal autopsy, sample collection and laboratory testing, entomology surveillance, social mobilization and reactive vaccination campaigns [18].

Active case search involved house-to-house visits in affected communities, where family members were quizzed on history of jaundice and fever within the period between July 1 and October 6, 2017. A similar search was carried out in all healthcare facilities in the affected communities by retrospective analysis of hospital records for patients with symptoms meeting the standard case definition. Individuals and patients who met these criteria were listed as suspected cases and the process gave rise to a total of 55 suspected cases [18]. A rapid yellow fever vaccination coverage assessment was also conducted to determine the yellow fever vaccination status of children aged 1–10 years old in the affected communities [18]. To achieve this, systematic sampling of alternate houses was done, starting from the meeting point of the RRT with the community and going in a clockwise direction. Members of the community were asked questions regarding the yellow fever vaccination status of the children and to provide evidence such as routine immunization cards, if possible [18]. The assessment result showed that 46% of children in all affected communities had been vaccinated for yellow fever, while only 27.5% could provide their immunization cards. In the hardest-hit Ifelodun LGA, only 25% of children were vaccinated [18]. The results of this assessment were used to draft a vaccine request to the ICG on vaccine provision, to support a reactive yellow fever vaccination campaign to follow.

A verbal autopsy was also carried out to determine the burden of morbidity and mortality, and assess for a potential under-reporting of yellow fever cases in the community. A case eligible for verbal autopsy was defined as: “any death of a family member(s) who before death developed acute onset of fever and jaundice with or without bleeding appearing within 14 days in a person who resided in Ifelodun or any other part of Kwara State between 1 July to 6 October 2017” [18]. The verbal autopsy revealed 26 deaths, 24 of which the RRT were able to sight their graves. All deaths were epidemiologically linked to a single case confirmed at the Institut Pasteur [18]. Testing involved collection of 5mL of blood sample from suspected cases for IgM serology and real-time polymerase chain reaction (RT-PCR) at the Central Public Health Laboratory, Lagos and the Lagos University Teaching Hospital (LUTH) virology laboratory. Confirmation tests were carried out at the Institut Pasteur, Dakar, Senegal, which serves as the WHO reference laboratory for the region [18]. Of the 55 cases uncovered by active search, 32 tests were carried out, yielding ten presumptive positive results and one inconclusive result, all of which were sent to the WHO regional reference laboratory, where seven were confirmed positive [18].

An entomological survey was conducted to confirm the presence of the yellow fever vector in the affected communities. The methods used for larval sampling include ovitraps and modified human landing catch (HLC), while for adult flies include: CDC UV light trap, BG-Sentinel trap, and CDC Light trap [18]. The survey findings demonstrated the presence of *Aedes* mosquito at various stages of development in six out of seven target communities. The vectors collected include the *Aedes africanus, Aedes aegypti* and *Aedes luteocephalus* species [18]. In-country next-generation sequencing of confirmed cases was also done at ACEGID, Ede, Osun state [45], to understand the genomic diversity and origin of YFV in Edo state.

The outbreak response also involved the activation of an IMS to coordinate a possible nationwide response. Through this IMS, other state MoH were notified to intensify surveillance in all states in the country. This was followed by the report of cases from other states such as Kogi and Zamfara [18]. Similar to Kwara, RRTs were deployed from the NCDC to these states, to help in outbreak response. The initial request for vaccines was made to the ICG on vaccine provision on September 26, 2017, and following the approval of the request, the first wave of yellow fever reactive vaccination campaign was carried out in the affected communities of Kwara state between the 11th and 20th October 2017. A second wave was implemented in two LGAs—Pategi and Edu between 7th and 8th December 2017 [18].

## 4. Limitations against effective infectious disease outbreak response

### 4.1. Rapid population growth

A significant factor to be considered while planning infectious disease preparedness and response strategies is the population for which these strategies are meant to serve. In the presence of rapid population growth, it would be expected that outbreak preparedness and response strategies also ought to evolve in a manner commensurate with the population increase, a lack of which would render outbreak preparedness and response strategies largely inadequate [29]. With a population growth rate ranging from 3.2% to 6.5% [46],[47] across the country, effective planning is made a herculean task for the public health agencies in the absence of much-needed resources and commitment from the political leadership to encourage a reduction in population growth.

### 4.2. Personnel shortage

A critical element necessary to mount a worthwhile infectious disease outbreak response is skilled human resources. The personnel working within different departments such as clinicians and nurses for case management, epidemiologists for contact tracing, and laboratory technicians for laboratory diagnosis and case confirmation are indispensable components in outbreak periods. It has been estimated that Nigeria has 27 doctors per 100,000 people [2], a statistic only about a quarter of the WHO recommendation of 100 per 100,000 for developing countries [48]. This shortage of physicians is representative of the rapidly dwindling number of frontline healthcare workers across all levels that pervades the Nigerian health system and has been blamed on the massive emigration of these personnel to other countries due to immense social and economic pressure and the search for greener pastures. Sadly, this is also true of the health systems of 36 other African nations [49]. Furthermore, losses as a result of retrenchment and restrictions on employment due to structural changes in the Nigerian health system have deprived the sector, but most importantly, rural communities, leaving them at the mercy of recurrent infectious diseases outbreaks and rendering outbreak preparedness and response efforts largely more reactive than proactive [2].

### 4.3. Poor healthcare funding

**Figure 2.**
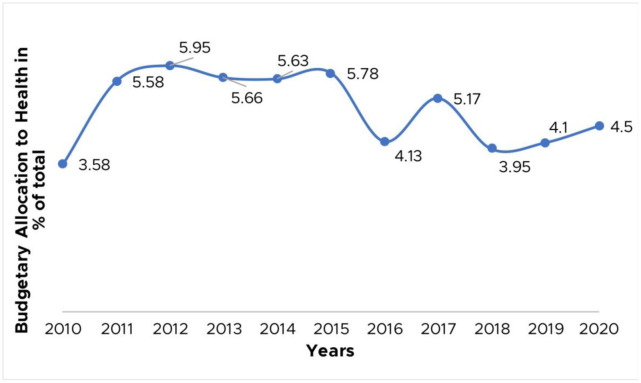
Nigeria's budgetary allocation to health 2010–2020.

Over successive years, the Nigerian health sector has been chronically underfunded, resulting in its widespread infrastructural deterioration, and a fall in the standards of health service delivery, invariably impacting negatively on outbreak preparedness and response strategies. In April 2001, African Heads of State under the umbrella of the African Union gathered in Abuja and resolved that each nation would commit a minimum of 15% of its annual budget to the development of its health sector [50]. It is however paradoxical that Nigeria, playing host to this “*Abuja Declaration*” has been unable to meet this target. Looking at the budgetary allocation to the health sector over the last 20 years (**Figure 2**), the highest percentage recorded has been in 2012 (5.95%), while the most recent figure for 2020 is a meager 4.5%, falling far short of the requirement of such an essential sector [51]–[53]. Consequently, in the midst of a shortage of much-needed resources, outbreak preparedness and response efforts are significantly hampered, as essential items such as PPEs for infection prevention and control are either not acquired or in perpetual shortage, while efforts towards investing in infrastructures such as isolation centers and diagnostic laboratories will be insufficient. Following an extensive search, we found that Nigeria had very little isolation capacity for infectious disease outbreaks prior to the onset of the ongoing COVID-19 pandemic. Apart from a few locations such as the Infectious Diseases Hospital, Yaba (115-beds) and the newly-opened Stella Obasanjo Hospital Isolation Centre, Benin (31-beds), there were no stand-alone infectious disease isolation facilities in the country, and the only ones that existed were all found in the southwestern region. The alternative to stand-alone infectious diseases isolation centers are infectious diseases wards of secondary and tertiary healthcare facilities, and a few other private establishments. However, the onset of the COVID-19 pandemic in Nigeria served as a catalyst for the creation of many stand-alone, albeit temporary isolation facilities all over the country, through several funding partnerships between the government and private sector players across various sectors.

### 4.4. Inadequate diagnostic capacity

In the wake of the 2014 EVD outbreak in West Africa, a report by the African Development Bank established the shortage in laboratory diagnostic capacity of many African countries, Nigeria inclusive [54]. As most infectious disease outbreaks are either viral infections requiring at least PCR laboratory capabilities or bacterial infections requiring cell culturing and susceptibility testing, it is imperative that an improvement of diagnostic capability be prioritized for effective outbreak preparedness and response strategies to be implemented. Underscoring this assertion is the fact that the availability of a virology laboratory at the LUTH, and genomic data made available by ACEGID, was critical to the rapid diagnosis of the index EVD case in Nigeria during the 2014 outbreak, allowing the early deployment of response efforts, which contributed greatly to Nigeria's success in curtailing the spread of the disease.

### 4.5. Political instability

Political instability, which is typical of many developing nations with young and fragile democratic systems such as Nigeria, creates a deplorable situation of inconsistency on outbreak preparedness and response strategies, resulting in long-term ineffectiveness of such efforts. According to a 2008 report by Kirigia and Barry [55], issues of leadership and governance such as inadequate healthcare legislation and weak enforcement of available healthcare laws, poor community participation in planning, management, and monitoring of healthcare services, health inequities, inefficient resource allocation, poor inter-sectoral collaborations and inadequate health information and research efforts have all contributed to the slow pace of development in the Nigerian health sector, immensely influencing outbreak preparedness and response efforts negatively.

### 4.6. Insecurity

Almost half of the West African countries have been classified as fragile states by the African Leadership Centre [56], battling one form of insurgency, political strife, or natural disaster. This labile state weighs heavily on public health response systems in these countries, contributing to a deterioration of existing healthcare infrastructure and limiting access to often life-saving healthcare services, making outbreak response in such settings challenging. In Nigeria's case, following a period of her removal from the list of nations endemic for the wild poliovirus in 2015, the smooth transition to being fully declared polio-free hit a temporary hurdle when three new cases were discovered in Borno, a situation attributed to the infrastructural collapse that followed the Boko Haram insurgency, ravaging the North-Eastern region at that time [24].

### 4.7. Absence of trans-border collaborations

The cross-border challenges experienced by Nigeria with her neighboring countries are a result of long-standing cultural, political, and economic relationships [57]. The three recent outbreaks of Lassa fever in Nigeria, Benin and Togo between 2017 and 2019 also revealed how critical information sharing could be to disease outbreak preparedness and response [58]. The absence of an adequate trans-border surveillance system increases the risk of infectious diseases being imported into the country through migrants.

### 4.8. Technological limitations

Information and Communications Technology (ICT) limitations such as gaps in internet connectivity and the dearth of ICT literacy among the population also limits the extent to which the internet is leveraged as a source of outbreak-related information [59]. On the other end, the internet has also been used as an effective tool of misinformation during the outbreak period, significantly hampering the efficacy of response efforts and causing heightened fear.

**Figure 3.**
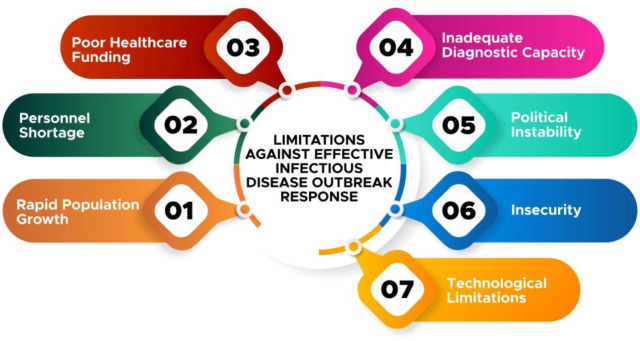
Overview of the limitations against effective infectious disease outbreak response in Nigeria.

An overview of the important limitations is described in **Figure 3**.

## 5. Implications and recommendations for policy and practice

### 5.1. On the economy

Liberia, Guinea, and Sierra Leone, the three countries worst hit by the 2013–2016 EVD epidemic in West Africa are estimated to have lost a combined USD 2.2 billion in Gross Domestic Product (GDP) in 2015 alone [60], beyond the mortality and social disruption experienced. Although Nigeria was able to curtail the pandemic in record time, it is estimated that at least USD 13 million was spent on mitigating the EVD outbreak in 2014 [61]. The World Bank's International Working Group on Financing Preparedness (IWG) has estimated that Nigeria could lose about USD 9.66 billion (approximately 1.98% of Gross National Income) per annum, due to the economic disruptions caused by the various infectious diseases outbreaks [60], while other WHO reports have established that it would cost just between USD 2.5 and 3.5 per person per annum to achieve infectious disease outbreak preparedness in the entire WHO African Region [62].

The NCDC also estimates that Nigeria needs NGN 139 billion (approximately USD 439 million) to implement her National Action Plan for Health Security (NAPHS) [61],[63], which addresses critical deficiencies in Nigeria's outbreak preparedness infrastructure, as identified by the WHO Joint External Evaluation (JEE) of International Health Regulations (IHR) core capacities of the Federal Republic of Nigeria [64]. It is thus clear that investing in outbreak preparedness is a far cheaper alternative to responding to disease outbreaks only after they have already occurred.

### 5.2. On disease surveillance

A study by Abubakar et al. [65] revealed that less than 50% of the criteria developed by the 2002 National Technical Guidelines for Integrated Disease Surveillance and Response (IDSR) implementation have been met in certain LGAs. As IDSR is the principal mechanism by which infectious disease outbreaks can be detected early and responded to effectively, more efforts are needed to fully implement the IDSR strategy, especially at the LGA level in Nigeria [66]. This can be achieved by prioritizing on-going training and capacity-building for local surveillance officers, especially in the areas of data collection, reporting, and improving access to laboratory diagnostic services. The use of mobile laboratories with capabilities to diagnose BSL 3/4 pathogens should also be considered. The deployment of BSL 3/4 mobile laboratories, particularly in resource-limited, remote areas, has the potential to reduce turnaround time, thereby facilitating better disease surveillance and patient management, reviewed by [67]. The onset of the COVID-19 pandemic has catalyzed the birth of several mobile BSL3/4 laboratories all across Nigeria, through several partnership agreements between the government and private sector organizations such as 54 gene, Aliko Dangote Foundation and Flying Doctors Nigeria Ltd. This has proven to be an effective strategy for increasing the diagnostic capacity of COVID-19 infections and contribute to the eventual flattening of the infection curve in the country. Private sector participation should also be strengthened at the local level, to improve access to much-needed resources for IDSR implementation. For sustainability, however, it is important that the IDSR funding is made a core part of the budget of various Federal and State MoHs.

### 5.3. On trans-border collaborations

The IHR capacities of Nigeria's neighboring countries such as Niger, Cameroon, Benin, and Chad are low, with each reporting at 44%, 38%, 35% and 25% capacity respectively [62]. This puts Nigeria at a high risk of being affected by epidemics arising from any of these nations, further reinforcing the need for effective collaborations with these countries, to develop regional capacities for disease preparedness, detection and response. Kakai et al. [58] went on to establish the need for increased surveillance of highly mobile and migrant populations, living near our borders, and acting as major vehicles of infectious diseases between neighboring countries, as a means of strengthening preparedness and response efforts.

### 5.4. On healthcare infrastructural development

Existing infrastructure and personnel from the Polio Eradication Initiative (PEI) were crucial in the 2014 EVD outbreak response in Nigeria and the West African region [68]. This demonstrates that horizontal integration of resources and capacities across different vertical disease response programs is possible, and could have an overall effect that strengthens health security. The core capacities of disease outbreak preparedness are heavily reliant on strong health systems and infrastructure, thus, funding should prioritize multi-use investments [69], such as communication infrastructure for quick dissemination of public health messages; investments in diagnostic laboratory capacities through upgrading the national reference laboratory, expansion of the NCDC molecular laboratory network, and development of state-level laboratories; implementing capacity building programs for key personnel (epidemiologists, health economists, biostatisticians, etc.); building of crucial health registries; as well as the acquisition and provision of vital medicinal supplies. These will substantially aid the outbreak preparedness and response efforts and strengthen the public health system. Furthermore, the use of geo-spatial technology that harmonizes data on geo-tagged healthcare facilities, population density and global travel patterns, can be used to identify which locations in the country have the least accessibility to healthcare facilities [70]. These analyses could also reveal which facilities are short of critical human and infrastructural resources and their potential to be badly hit by infectious disease outbreaks. These could also inform decisions on where new healthcare facilities should be cited to achieve maximum population coverage and impact across the country.

### 5.5. On outbreak preparedness and response coordination

In order to develop preparedness strategies for infectious disease outbreaks caused by known pathogens, it is important to develop protocols to guide ongoing preparedness and eventual response efforts. This can be achieved by identifying the trigger factors for the different components of the public health system, and by sectioning preparedness and response strategies into specific phases with detailed descriptions of the activities to be implemented in each phase [71]. Such protocols should also include strategies to coordinate between public health and clinical health services, promote private sector involvement, and provide regular situation updates to all stakeholders, thus ensuring prompt resource mobilization for outbreak preparedness and response efforts as well as prevent wastage at the onset of each outbreak. In addition, there is a need to develop protocols to guide post-epidemic actions as well as pre-emptive steps to be implemented in between outbreaks [72].

These post-outbreak efforts should begin with community engagement to understand the beliefs and perceptions of the public about the disease, which would in turn inform efforts towards educating the public as necessary. In addition, the importance of sanitation and hygiene should be communicated to the public, and they should be made aware of the fact that hygiene recommendations are not to be abandoned after one outbreak is curtailed, only to be revisited at the start of another. It is important that all these recommendations be implemented in a phased manner, involving all levels of the public health system: from the primary healthcare facilities in the LGAs all the way to the secondary and tertiary healthcare facilities. It is also crucial that these changes be coordinated by the State MoH, with the NCDC and the Federal MoH having an oversight function.

### 5.6. On intersectoral collaborations

The media plays a critical role in providing accurate and up-to-date information about disease outbreaks, thus aiding community mobilization and disease surveillance and helping to dispel fear and debunk myths among the general population and even among healthcare workers. A study has recently shown that 72% of healthcare workers in the Ashanti region of Ghana indicated the radio as their main source of information during the EVD epidemic [73]. Furthermore, collaborations and joint training with the police and other law enforcement agencies could be critical during periods of insecurity and civil unrest which may distort public health services such as contact tracing, disease surveillance and immunization efforts as seen in the case of the wild poliovirus (WPV) outbreak in Borno state [74],[75].

### 5.7. On quality control strategies

Another area of concern found by the WHO JEE mission to Nigeria is the lack of conduct of regular simulation exercises to improve capacity and identify the gaps in public health response to infectious disease outbreaks [64]. The first of such simulations was conducted one year after this report, in 2018, which was a full simulation of a yellow fever outbreak [76]. Simulation exercises also help to assess the effectiveness of public health partnerships. Many more of such simulations need to be done going forward, and with as little foreknowledge of participants as possible.

### 5.8. On political leadership and governance

**Figure 4.**
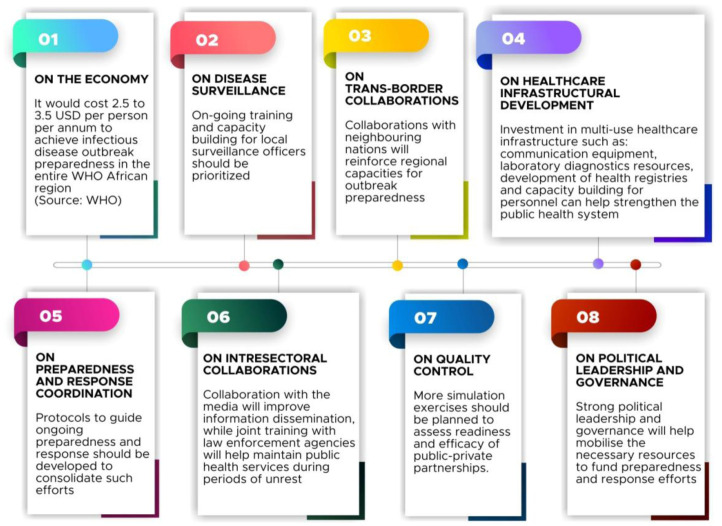
Overview of implications for policy and practice.

Since its establishment in 2011, the NCDC has assumed the responsibility of coordinating the response to disease outbreaks in the country, producing commendable results. The approval of the NAPHS in 2018 [61] and the passing of the NCDC executive bill in the same year [77] were important initial steps towards the implementation of the action plan. Having developed a financial plan, the NCDC should push for the incorporation of the NAPHS funding into core components of the national budget to ensure sustainability. To achieve this, it may be beneficial to partner with the Ministry of Finance and other relevant agencies to determine ways of mobilizing the needed resources to finance preparedness and response strategies [60]. Also, strong political leadership and governance is a necessity if the NAPHS is to be implemented successfully in Nigeria. Regrettably, however, we have already fallen behind the deadline for scheduled assessments on the progress of the implementation, which ought to happen at intervals of six months. When such reports are eventually compiled, it is important that they be made publicly available for accountability sake. **Figure 4** summarizes the key policy recommendations discussed above.

## 6. Conclusion

Following an extensive review of the available body of evidence, it would seem that Nigeria is indeed going in the right direction as regards infectious disease outbreak, preparedness, and response, albeit not at the required pace. The Nigerian story with infectious disease outbreaks does prove that with the right structure and with local public health institutes like the Nigerian Center for Disease Control (NCDC), even low-resource countries stand a chance against the deadliest of infectious diseases outbreaks. However, this structure must be complemented with the appropriate personnel, sincere commitment from the political leaders and foresight to plan for even the unlikeliest of situations, as no efforts spent towards infectious disease outbreak preparedness is a waste. Even during the on-going COVID-19 pandemic, many of the infrastructure and personnel that were developed towards an effective response to Poliomyelitis and Ebola Virus Disease, have proved to be instrumental to Nigeria's response to the new foe. For example, the first case of COVID-19 in Nigeria was diagnosed at a laboratory facility set-up during the EVD outbreak of 2014. The prioritization and implementation of the National Action Plan for Strengthening Health Security (NAPHS) would accelerate national progress towards the development of efficient public health infrastructure to facilitate adequate preparedness and response efforts.
